# POEMS syndrome: Two cases for the general physician

**DOI:** 10.1016/j.clinme.2025.100506

**Published:** 2025-08-25

**Authors:** Zoe Maikovsky, Peter Williams

**Affiliations:** University Hospitals Sussex, Worthing Hospital, Lyndhurst Road, Worthing BN11 2DH, UK

**Keywords:** POEMS syndrome, Paraneoplastic syndrome, Atypical ischaemic stroke, General physician, Rare disease

## Abstract

Set in a district general hospital, this case series explores two individuals who developed a rare multisystemic syndrome:; polyneuropathy, organomegaly, endocrinopathy, monoclonal gammopathy and skin abnormalities (POEMS). Diagnostic journey, trajectory of disease and outcomes are compared. Both patients presented to healthcare numerous times and saw multiple specialists for symptoms resulting predominantly from volume overload and neuropathy, prior to being admitted with atypical ischaemic stroke. During their admission, diagnosis was made after atypical intracranial arterial stenoses, sensory neuropathy not in keeping with their stroke, plasmacytoma detection and confirmatory raised vascular endothelial growth factor (VEGF) levels. POEMS is highly treatment responsive, survival rate improving with earlier diagnosis. Both patients were transferred to specialist centres for chemotherapy. Unfortunately, patient outcomes significantly differ, one having favourable recovery, while the other experiencing treatment-resistance disease requiring local repatriation for withdrawal of care. We identify challenges faced by both patients and the medical team, and discuss the importance of the general physician within the world of specialist medicine, in order to provide holistic, comprehensive patient care.

**Key learning points.**

• Features of polyneuropathy, paraproteinaemia, skin haemangiomas, fluid overload.
• A potential cause of global exudative, extravascular fluid overload.
• In subacute stroke with polyneuropathy, send highly specific VEGF levels.
• Multisystem disease requires close cross-specialist working within ambulatory care.
• Highly treatment responsive; chemotherapy, radiotherapy and stem cell transplant.

Alt-text: Unlabelled box

Key to diagnosing POEMS (polyneuropathy, organomegaly, endocrinopathy, monoclonal gammopathy and skin abnormalities) is recognition of proinflammatory and prothrombotic states combined with monoclonal gammopathy and skin lesions.

This rare paraneoplastic syndrome^1^ is related to proinflammatory cytokines, including vascular endothelial growth factor (VEGF). Presenting more frequently in men, median onset sixth decade,^2^ with multisystemic features (Fig. 1).^3^ The 10-year survival rate is 80%;^4^ however, many cases go undiagnosed. Correct diagnosis is essential to providing definitive treatment with chemotherapy, radiotherapy and haematopoietic cell transplantation.

**Fig. 1:**
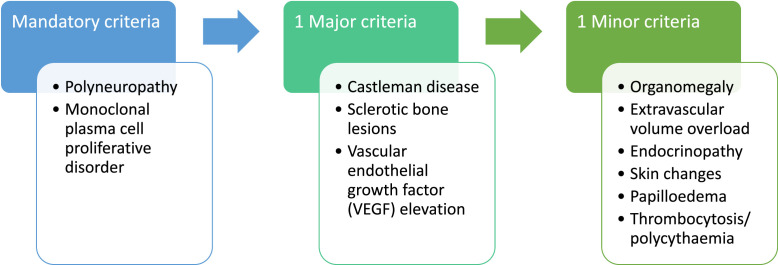
**Diagnostic criteria [3], requiring both mandatory criteria, one other major and one minor criteri**on**.** Description: flow diagram, illustrating key criteria required for diagnosis.

## Case presentation

Case 1: a 36-year-old man presented twice to his GP; initially with weight loss, myalgia, abdominal pain and gynaecomastia; subsequently with bilateral leg swelling and diarrhoea. Liver function tests were deranged and ultrasound revealed coarse liver parenchyma, splenomegaly, ascites and pleural effusions. Initial differential was of liver disease, and spironolactone was trialled to manage suspected anasarca. At 5 months, he presented to hospital with weight loss, abdominal pain, exertional breathlessness and leg swelling. Gastroscopy, to investigate his unexplained weight loss, revealed *Helicobacter pylori* gastritis (subsequently treated). CT and paracentesis demonstrated exudative ascites, pleural effusion and lymphadenopathy.

At 7 months, his abdominal pain worsened and he developed left-sided paraesthesia. Raised protein–creatinine ratio, paraproteinaemia with raised IgG and severe pancreatic and adrenal insufficiencies were detected, and Creon supplementation initiated. Initial bone marrow biopsy was inconclusive, ultrasound visualised reactive lymphadenopathy and brain MRI revealed hypoperfusion lacunar infarcts. He was not treated for stroke at this stage and, on discharge, a unified diagnosis was not made. Dieticians noted a 5-stone weight loss at 8 months, despite a 3,000 kcal daily intake.

At 11 months, he was admitted with left arm weakness, intermittent visual disturbance and skin lesions over the torso and limbs. Differentials included vasculitis and amyloidosis, treated with steroids. There was no amyloidosis on cardiac MRI. Hemiparesis progressed and partial seizures developed. Skin lesions were biopsied. Brain MRI demonstrated right parietal infarct with anterior cerebral artery stenosis. Due to concern of underlying malignancy or multisystem amyloidosis, with persistently raised CRP and potential paraneoplastic phenomena, PET CT was performed. This elucidated a large left pelvic plasmacytoma, biopsied in tertiary orthopaedic unit. Based on this finding, together with serum VEGF of 18,577 pg/mL, POEMS was diagnosed. He was transferred to a London specialist centre (LSC) for 5 months of chemotherapy (velcade and dexamethasone). Recovery began, he gained weight and seizures reduced. Initially discharged on aspirin and apixaban, the latter stopped after VEGF reduced to 950 pg/mL a year later.

**Fig. 2:**
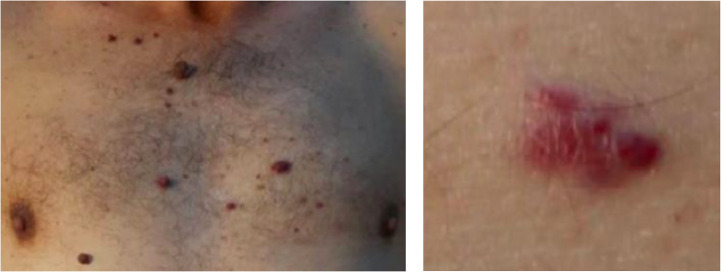
**Images of glomeruloid haemangiomatous lesions from patient 2**. Description: photographs of skin lesions, the left demonstrates distribution over the chest, the right focuses close up on detail from a singular lesion. Permission given by spouse of patient.

Case 2: a 55-year-old man with hypertension, hypercholesterolaemia, type 2 diabetes mellitus and epilepsy. He first presented to GP with erectile dysfunction, suspected benign and was prescribed sildenafil. He attended hospital after dropping items and mobility worsening and demonstrated reduced grip strength, paraesthesia of both feet and right arm, and peripheral oedema. Differential diagnoses included neuropathy or myositis, then later Guillain–Barré syndrome and multiple sclerosis. Blood tests revealed raised BNP at 1,012 pg/mL (serum autoantibodies, viral hepatitis, HIV, CMV and EBV screens all negative; HbA_1C_ and B12 normal; folate mildly low). Brain CT and MRI were unremarkable; spine MRI showed mild discopathy. Ambulatory care noted ongoing right hand weakness, new right facial numbness and progressive skin lesions over forehead and torso (Fig. 2). Progressive right upper limb mild motorsensory neuropathy continued.

At 4 months, he presented to hospital with 2 weeks of intermittent speech disturbance, right facial droop and diplopia. Imaging was not repeated due to recent MRI; however, kappa and lambda free light chains were raised with normal ratio. Soon after, he reported worsening breathlessness and was found to have pleural effusions (worse on the left). Malignancy was suspected after 1.5 L of exudative fluid was drained, although cytology returned negative.

Later that month, he presented acutely with right inattention, dysarthria and right-sided hemiparesis (NIHSS 7). CT revealed established infarcts of the left frontoparietal lobes in conjunction with terminal ICA, MCA and ACA stenoses. Staggered presentation and multivessel disease raised suspicion of vasculitis. Lumbar puncture to exclude encephalitis was delayed due to bleeding risk after starting steroids and early dual antiplatelets. Skin biopsies, VEGF of 5,385 pg/mL and discovery of a right iliac expansile lytic lesion on PET CT led to POEMS diagnosis. LSC guided local management while awaiting transfer. Sadly, deterioration required intensive care support for seizure management due to evolving cerebral infarctions despite heparin and aspirin therapy, complicated by significant non-intracranial bleeding events. In that short time, VEGF surged to 15,355 pg/mL and he transferred to LSC for chemotherapy with bortezomib, daratumumab and dexamethasone. On arrival, prognosis was poor; however, given POEMS’ treatment response, chemotherapy was attempted. VEGF rose rapidly, suggesting resistant disease. A month later, he was repatriated for withdrawal of care closer to relatives.

## Discussion

**Fig. 3:**
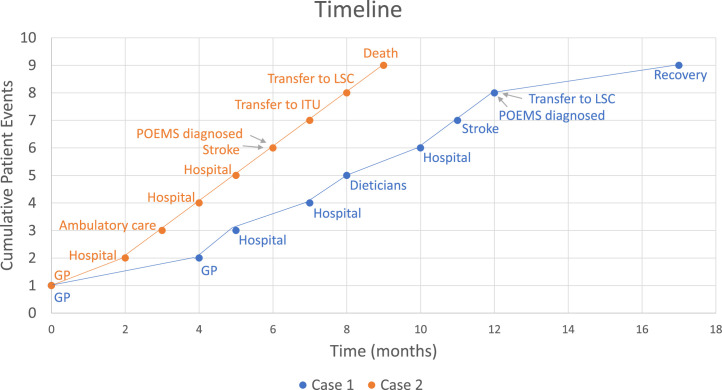
**Timeline of patient events** Description: line graph of presentations to health care, stroke onset, POEMS diagnosis, transfer to London specialist centre (LSC) and outcome.

Both cases describe an initial prodrome of progressive, non-specific symptoms at which time diagnosis is challenging, followed by rapid deterioration. Repeated presentations (Fig. 3) are not uncommon; this trajectory typically delays diagnosis by 12–16 months.^5^ Our patients had multiple specialist reviews prior to diagnosis, including respiratory, rheumatology, dermatology, endocrinology, stroke and haematology. Case 2 highlights the brief window between clinical onset and rapid decline – prompt recognition is critical for timely chemotherapy. POEMS is highly treatment responsive, with earlier commencement improving survival; in 2 decades, 5-year overall survival in high-risk patients has risen from 60% to 92%.^5^

**Fig. 4:**
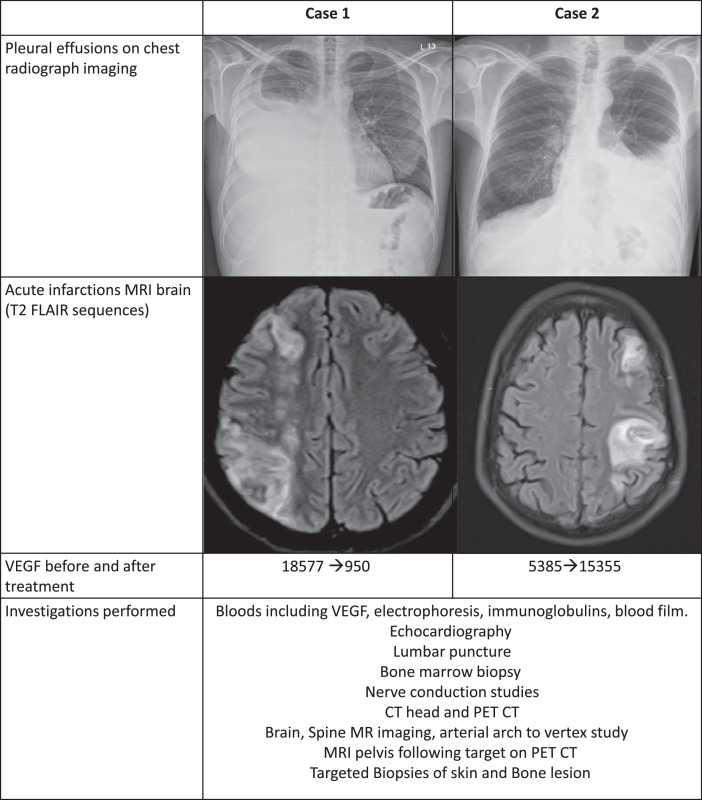
**Investigations for each case** Description: table describing investigations performed and key results including chest radiographs and MRI images.

Ischaemic events from atypical intracranial artery stenosis marked the turning point for diagnosis (Fig. 4), together with plasmacytoma detection and pathognomic cutaneous glomeruloid haemangiomas. These lesions, present in 65% of cases,^6^ may help earlier disease recognition. Myelomatous paraproteinaemia, another common feature, was found in our cases with elevated IgG, kappa and lambda free chains. This perhaps explains POEMS’ highly prothrombotic nature (30% sustaining arterial or venous thromboembolism^7^), as demonstrated by our patient 2, who acquired further infarcts despite combined antithrombotics.

Our cases illustrate multisystemic manifestations of POEMS, its trajectory, diagnostic challenges for clinicians and patients, and need for multi-specialist collaboration to coordinate care. VEGF is useful in diagnosis, with 100% sensitivity and 90% specificity.^8^ The disease is highly treatment responsive. Earlier diagnosis improves survival and reduces morbidity from stroke.

## Ethics approval and consent to participate

This article has not been reviewed by an ethics committee.

## Consent

1. Patient 1 has given written consent for publication of a case report.
2. Patient 2 gave consent when photographs were taken for this to be added to his notes and for possible educational use. He unfortunately died before we were able to ask for consent to write a case report. We have sought written consent from his wife on his behalf, who agrees for a published case report and images of skin lesions being used.

## CRediT authorship contribution statement

**Zoe Maikovsky:** Writing – original draft, Project administration, Formal analysis, Data curation. **Peter Williams:** Writing – review & editing, Supervision, Conceptualization.

## Declaration of competing interest

The authors declare that they have no known competing financial interests or personal relationships that could have appeared to influence the work reported in this paper.
